# Peptide profiling in cow urine reveals molecular signature of physiology-driven pathways and in-silico predicted bioactive properties

**DOI:** 10.1038/s41598-021-91684-4

**Published:** 2021-06-14

**Authors:** Rohit Kumar, Syed Azmal Ali, Sumit Kumar Singh, Vanya Bhushan, Jai Kumar Kaushik, Ashok Kumar Mohanty, Sudarshan Kumar

**Affiliations:** ICAR-National Dairy Research Institute, Cell Biology and Proteomics Lab, Animal Biotechnology Center (ABTC), Karnal, Haryana 132001 India

**Keywords:** Mass spectrometry, Proteomic analysis, Peptides, Proteases, Urological manifestations

## Abstract

Peptidomics allows the identification of peptides that are derived from proteins. Urinary peptidomics has revolutionized the field of diagnostics as the samples represent complete systemic changes happening in the body. Moreover, it can be collected in a non-invasive manner. We profiled the peptides in urine collected from different physiological states (heifer, pregnancy, and lactation) of Sahiwal cows. Endogenous peptides were extracted from 30 individual cows belonging to three groups, each group comprising of ten animals (biological replicates n = 10). Nano Liquid chromatography Mass spectrometry (nLC-MS/MS) experiments revealed 5239, 4774, and 5466 peptides in the heifer, pregnant and lactating animals respectively. Urinary peptides of <10 kDa size were considered for the study. Peptides were extracted by 10 kDa MWCO filter. Sequences were identified by scanning the MS spectra ranging from 200 to 2200 m/z. The peptides exhibited diversity in sequences across different physiological states and in-silico experiments were conducted to classify the bioactive peptides into anti-microbial, anti-inflammatory, anti-hypertensive, and anti-cancerous groups. We have validated the antimicrobial effect of urinary peptides on *Staphylococcus aureus* and *Escherichia coli* under an in-vitro experimental set up. The origin of these peptides was traced back to certain proteases viz. MMPs, KLKs, CASPs, ADAMs etc. which were found responsible for the physiology-specific peptide signature of urine. Proteins involved in extracellular matrix structural constituent (GO:0005201) were found significant during pregnancy and lactation in which tissue remodeling is extensive. Collagen trimers were prominent molecules under cellular component category during lactation. Homophilic cell adhesion was found to be an important biological process involved in embryo attachment during pregnancy. The in-silico study also highlighted the enrichment of progenitor proteins on specific chromosomes and their relative expression in context to specific physiology. The urinary peptides, precursor proteins, and proteases identified in the study offers a base line information in healthy cows which can be utilized in biomarker discovery research for several pathophysiological studies.

## Introduction

Excretory biological fluids such as urine, saliva, milk, tear, mucus and sweat are significantly important for the maintenance of homeostasis in normal physiological conditions. Urine being a glomerular filtrate of blood is capable of summarizing the events that occur in the body as a result of changing physiology or pathological conditions. The systemic changes are well reflected by qualitative and quantitative alterations in the urine composition. No wonder, urine has been considered as excellent sample for the discovery and detection of biomarkers associated with general health and disease. Endogenous peptides and proteins secreted in urine have proven as hallmarks of various pathophysiological changes and have emerged as a better option over other biological fluids as it can be obtained in a large volume without much maneuver in dairy animals.

The quantitative estimation of urinary biomarkers is affected by changes in the volume of voided urine^1^. In the absence of a standard baseline profile of peptides in the urine, identification and quantitation of biomarkers usually becomes challenging. The peptides present in urine remain stable for a long time. The process of degradation of urinary proteins into resultant peptides is completed by the time of sample collection, whereas protease activation in fluids like the serum, plasma, and saliva remains continued even after sample collection that affects the actual result^2, 3^.

Besides, the metabolites, minerals, and salts in the glomerular filtrate, urine is composed of peptides and proteins originating from tubular secretion, secreted exosomes, and epithelial cells shed from the kidney and urinary tract^4^. Urine carries more local information as indicated from studies that showed that 70% of the proteome comprises secretions down from the kidney to urinary tracts and the remaining 30% originates from plasma^5^. Under the Human Kidney and Urine Proteome Project, comprehensive studies has been carried out to find novel biomarkers associated with kidney diseases, and to unravel the proteome of urine in normal and diseased conditions^6–9^. Extensive investigation of human urinary endogenous peptides has been carried out in the context of various diseases stating their strong clinical relevance^10–12^. However, studies in bovine are still in the early phase. Around 1550 proteins have been identified in the urine of healthy Karan fries cows^13, 14^. Although reports related to protein-based biomarkers in saliva and urine are present^14–16^, the peptidome based studies are very scanty in bovine and most of the studies belong to milk. Milk has been explored for its peptide and protein profiling, identifying around 8559 unique peptides and 6210 proteins, respectively^17, 18^. Lack of studies in bovine urinary peptidome makes it difficult for researchers to explore and investigate urine for its application in diagnosis and clinical application. A comprehensive urinary peptidome database encompassing diverse peptides tracing back to systemic and local origin which are processed by a variety of endogenous proteases can tell a defined story of animal physiology which can be referred to in the future for studies involving bioactive peptides, and biomarker discovery for clinical diagnosis and prognosis.

Although contemporary studies are inclined towards the discovery of biomarkers associated with a particular condition or disease, the data generated by MS/MS analysis of urine can track the changes in the proteome & peptidome not only in a condition-specific manner but also in establishing physiology-specific bio-molecular signatures present in it. In that direction, the foremost thrust in the field of clinical proteomics is the profiling of such endogenous peptides.

The current study reveals urinary peptidome in the Sahiwal breed of cows across different physiological stages and their resourcefulness for various applications. Urine samples were collected from 10 healthy animals in each group, namely heifer, pregnant and lactating. The peptides were identified and characterized to signify its importance in deriving meaningful information in the context of physiological changes and several other attributes associated with cow urine.

## Results

### Endogenous peptide profile in cow urine

**Figure 1:**
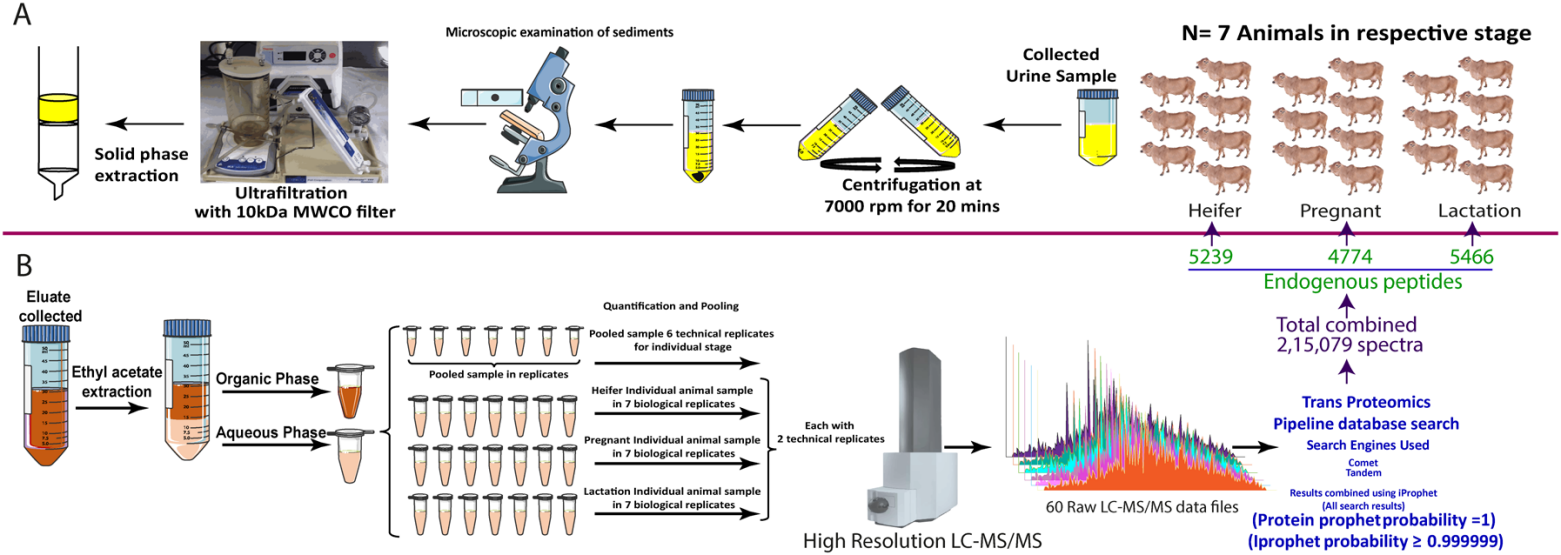
(**A**) Flow diagram showing collection and processing of samples from three different physiological states of Sahiwal cow followed by separation of small-sized peptides (<10 kDa) by ultrafiltration assembly and its extraction by C18 solid-phase extraction method. (**B**) The solid phase extraction (SPE) method. The bound analytes in the column were first eluted in  60% ACN, 0.1% TFA followed by the extraction of peptides in the aqueous phase using ethyl acetate. An equal amount of peptides from each sample (biological replicates) was pooled group-wise and quantified, for nLC-MS/MS analysis in triplicate (technical replicates).

The endogenous peptides were purified using a solid-phase ethyl acetate extraction method (Fig. 1A). The total collection of physiologically distinct (heifer, pregnant, and lactation) urine samples resulted in 2,15,079 spectra (Fig. 1B). The pooled samples (n = 10) in each physiological state resulted in the identification of 5239, 4774, and 5466 endogenous peptides in the heifer, pregnant, and lactation, respectively. The dataset was analyzed using Trans Proteomics Pipeline (TPP) with three independent search engines. The complete results summary has been provided in Table 1. The protein prophet and iProphet value of 1 and 0.9999 respectively, along with less than 1% FDR was used as a cutoff to select the highly confident peptides (Supplementary Table 1) (Fig. 1B). Initial screening of mapped peptides showed the highly diversified nature of the endogenous peptidome.

**Table 1:** # Total number of unassigned spectra acquired in all the raw data mapped in COMET search engine using no enzyme search parameter.

Total raw files	Conditions	Unassigned total spectra#	Sum total spectra (after assignment)	Endogenous De novo Peptides	Proteins (Protein prophet probability=1) (Iprophet probability$\ge$0.999999)
20	Heifer	214471	27083	5239	2092 (900)*
20	Pregnant	218556	27336	4774	2002 (755)*
20	Lactation	212208	27025	5466	1946 (860)*

*Proteins reported with bold letters asterisk symbol are identified with more than 10 percent sequence coverage peptides of respective protein and all the peptides identified through minimum of 2 independent spectra.

### Molecular characteristics of urinary peptides

**Figure 2:**
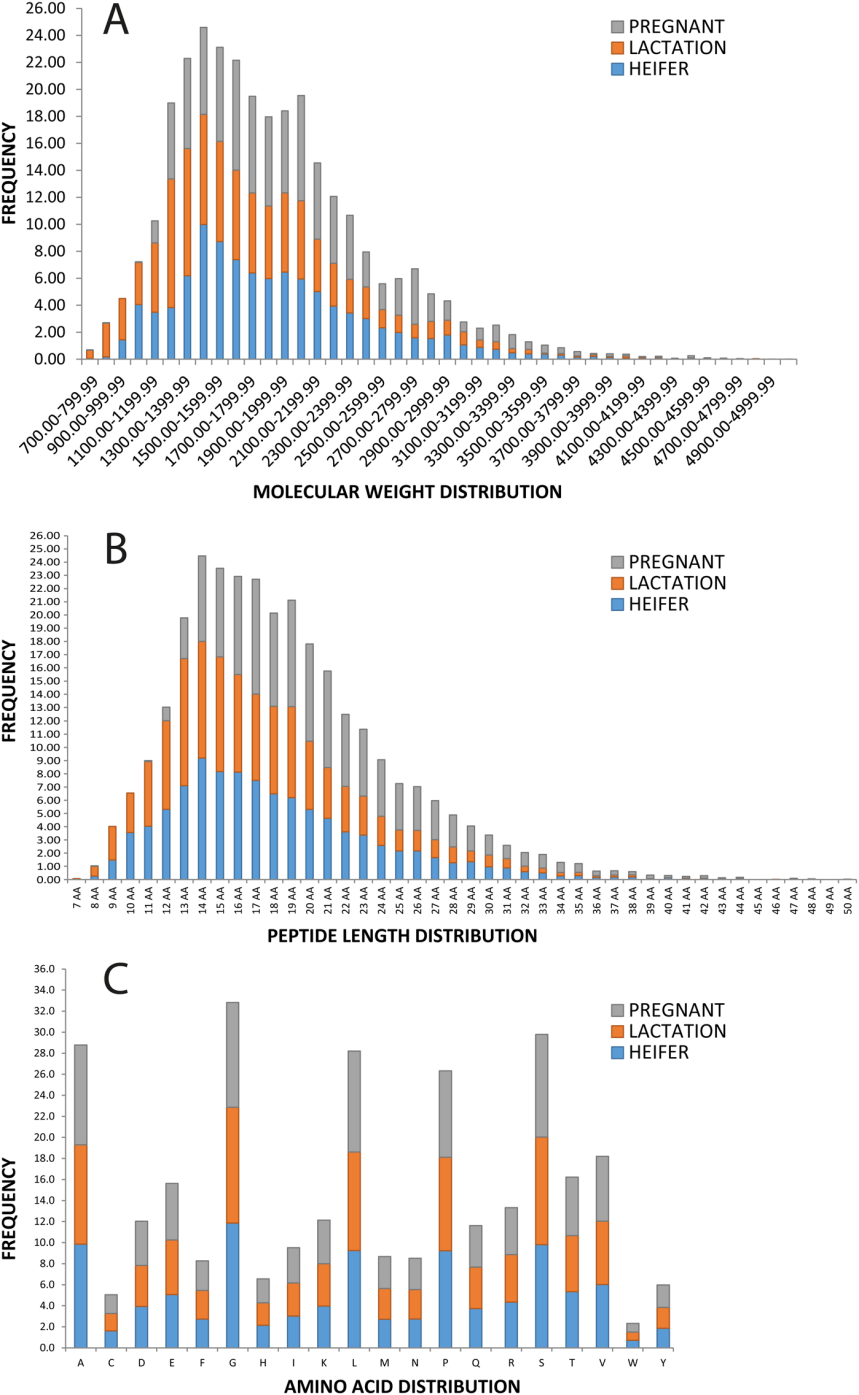
(**A**) Distribution of molecular weight of peptides along with their relative abundance. (**B**) Peptide length distribution and (**C**) Distribution of amino acid residues in three different groups.

**Figure 3:**
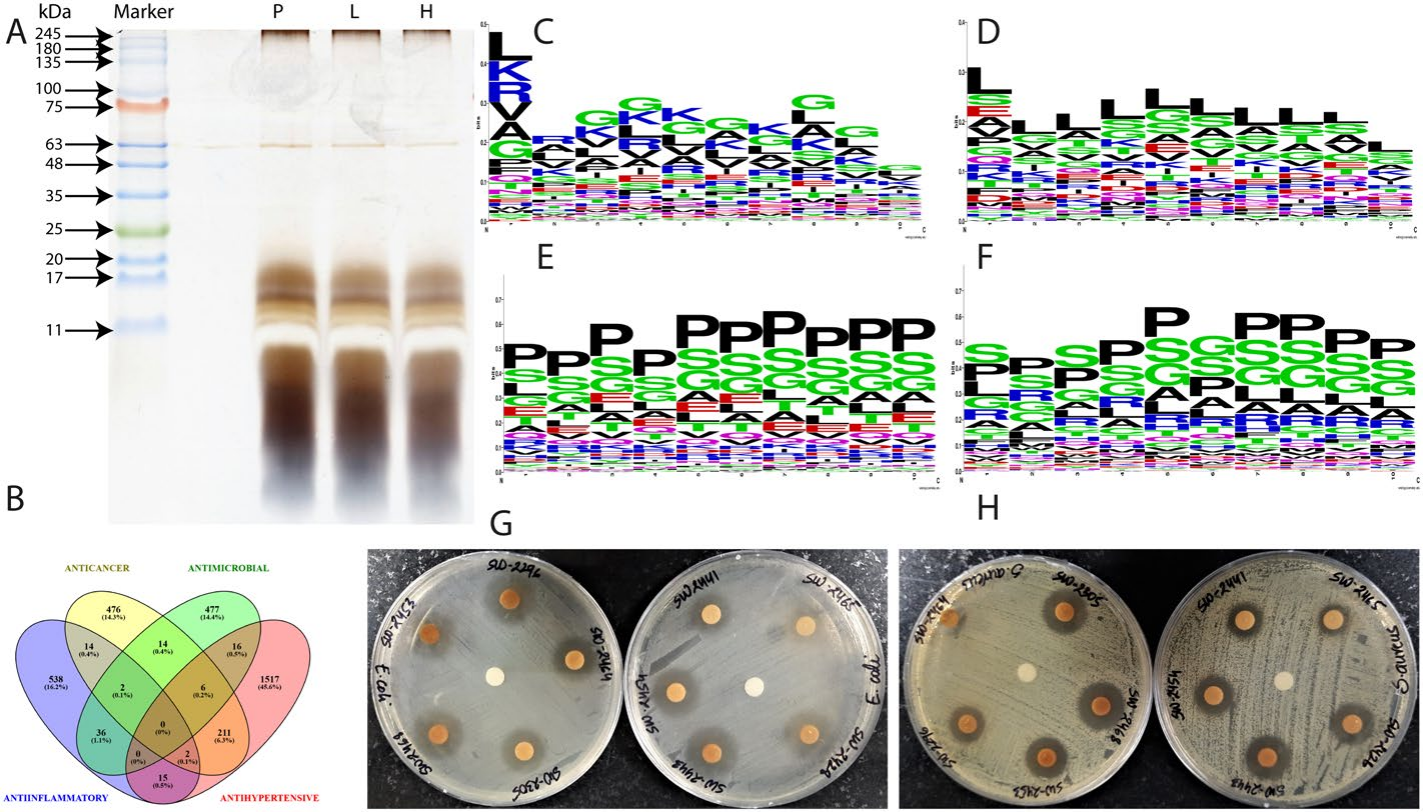
(**A**) After subjecting <10 kDa cow urine fraction to Solid Phase extraction, the eluate was collected in 60% ACN, 0.1% TFA. Ethyl acetate extraction of the eluate was carried out and the aqueous phase was separated and dried in Speed Vac. Aqueous extract pooled from different physiological states viz. Heifer, Lactation, and Pregnant (left to right) of Sahiwal cattle were then visualized by tricine-SDS PAGE. (**B**) Venn diagram showing the number of common peptide sequences shared by different classes of predicted bioactivity. (**C**) N terminal (10 amino acids) sequence logos were generated for the sequences predicted with certain bioactivity. Antimicrobial Peptide sequences showed glycine, lysine, and leucine as dominant residues. (**D**) Sequence logo for predicted Anti-Inflammatory sequences, every position is predominated by leucine residue. (**E**) Sequence logo for predicted Antihypertensive sequences with Proline as dominant residue. (**F**) Sequence logo for predicted Anticancer sequences with Proline as dominant residue. Both antihypertensive and anticancer sequences show proline as the dominant amino acid at almost every position. (**G**) 20 µL of Solid Phase Extract eluate from 10 individual animals (Heifer) was coated on 6mm sterile disc and placed on Staphylococcus aureus lawn (equivalent to 0.5 McFarland units). Zone of Inhibition was observed after 12–18 h of incubation. Disc in the center is a negative control containing 20 µL of BSA digest. (**H**) Peptide extracts activity against Escherichia coli (equivalent to 0.5 Mc Farland). Disc in the center is a negative control containing 20 µL of BSA digest.

The frequency distribution of molecular weight, peptide length, and amino acid composition of peptides belonging to three different groups have been shown in (Fig. 2). The peptide from different groups were found to have a molecular size of less than 10 kDa (Fig. 2A). In heifer and lactating animals’ urine, low molecular weight peptides ranging from 1.4–1.5 kDa were more prevalent. In contrast, in pregnant animals’ urine, peptides of relatively large size in the range of 1.8, 2.2, and 2.9 kDa were more prevalent (Fig. 2A). The peptides length distribution comparison showed equal frequency in three conditions (Fig. 2B). However, amino acid composition indicated that alanine, glycine, leucine, proline, and serine (in decreasing order of abundance) were the most frequently occurring amino acids (Fig. 2C) which reflected a similar pattern across the three physiological states under study. The band pattern of peptides in Tricine-gel showed similar results across different groups (Fig. 3A). The present study focused on endogenous peptides by filtering out the molecules larger than 10 kDa using a 10 kDa cut-off membrane. The observation agreed with the findings of molecular characteristics (Fig. 2) that the urinary peptides are of low molecular weight, i.e., less than 10 kDa. In summary, the results suggest that endogenous peptides in urine are highly diversified in sequences but contain comparable molecular characteristics. It guided us to identify the cause and origin and impact of underlying diversity in the peptidome.

### Functional attributes associated with urinary peptidome

Urine is full of secretions which offer protection to the urogenital tract from the environmental pathogens. We characterized urinary peptides with several biological activities using in-silico and in-vitro experiments. In-silico experiments were conducted to classify the bioactive peptides into anti-microbial, anti-inflammatory, anti-hypertensive, and anti-cancerous groups. The notion was to identify the mass spectrometer-based profiled cow urinary peptides in all the classes and to narrow down the list of peptides to a few significant candidates which exhibited wide spectrum of activities. Excitingly, we identified 15 peptides displaying in-silico activity of all types of processes (Fig. 3B). Anticancer and antihypertensive sets shared 211 peptides constituting 6.3%. The antimicrobial (n = 551) and anti-inflammatory (n = 607) peptides shared 36 sequences (Fig. 3B). Sequence analysis of all the peptides showed that glycine amino acid was a common and most abundant residue in all the classified peptides (Fig. 3C, D, E, and F). This bears an important message in the designing and synthesis of broad-spectrum bioactive peptides that can perform multiple functions.

A total of 551 high scoring antimicrobial peptide sequences were used to determine the consensus motif of amino acids. The average hydrophobicity in the sequences was −0.13 with an average charge of +1.8, indicating that sequences are hydrophilic and cationic. The sequence logo showed the dominant presence of positively charged residues like Arg and Lys, whereas Gly and Leu were dominant hydrophobic residues (Fig. 3C). A total of 607 sequences were predicted to possess anti-inflammatory activity. A positional conservation study showed that leucine residue was relatively dominant at the first 10th position from the N-terminal end in all anti-inflammatory peptides. Positional conservation study at N-terminal of anti-inflammatory peptides showed Leu, Tyr, Ser, Arg, and Glu to be highly conserved residues. While its position may vary, Leu residue seems to be central to the anti-inflammatory activity (Fig. 3D). We identified 1767 sequences predicted with antihypertensive activity with Pro as dominant residue at all positions (Fig. 3E). In comparison, 724 unique sequences were predicted to possess the activity mentioned above (Fig. 3F) (Supplementary Table 2). For validation of the in-silico finding, we tested the antimicrobial activity of isolated peptides against two pathogens, *E. coli* and *S. aureus* (Fig. 3G and H). The pure peptide extract showed a significant zone of inhibition in the disk diffusion method with a mean inhibition zone of $1.22\pm 0.11$ (SD) cm; and SEM: $\pm 0.03$ cm on *E. coli* and mean inhibition zone of $1.22\pm 0.10$ (SD) cm; SEM: $\pm 0.03$ cm) on *S. aureus*. The results of in-vitro experiments suggest that urinary peptides possess strong antimicrobial activity.

### Bovine urine degradome

**Figure 4:**
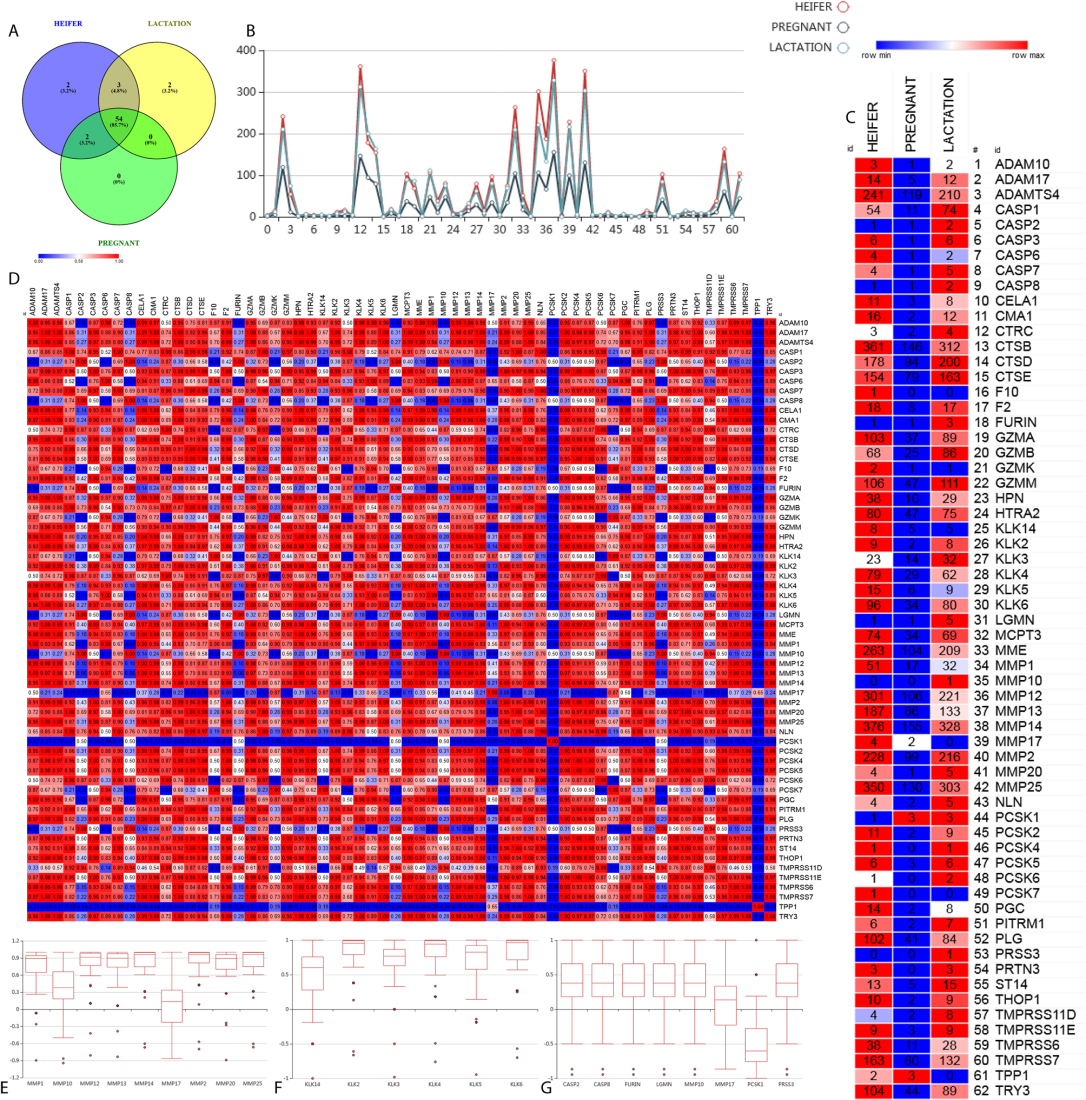
Degradome Analysis: (**A**) Venn Diagram describing the common and unique protease enzyme identified among the stages in the protein data mapping. (**B**) Profile plot depicting the quantitative values of target proteins (Y-axis) map to 62 different protease enzymes (X-axis). (**C**) Quantitative heat map presenting the protease enzyme class with the respective target number in three conditions. (**D**) Pearson correlation-based similarity matrix plot of all protease enzymes and quantitative target relationship. (**E**) Distribution of isoforms of MMP enzymes identified in protein mapping and respective target determined in peptidome. (**F**) Distribution of isoforms of KLK enzymes isoforms identified in protein mapping and respective target determined in peptidome. (**G**) Protease wise comparison for Pearson correlation of 8 different enzyme (CASP2, CASP8, FURIN, LGMN, MMP10, MMP17, PCSK1, and PRSS3) in disagreement with physiological state and uncorrelated with one another.

Manual curation of 22 selected proteases resulted in the discovery of an average of 7215 protease sites (Supplementary Table 3). The urinary peptides were investigated for four residues either from N or C terminal and peptides were sorted based on possible protease that might be involved in its release from the precursor protein. We found physiology-driven variation in the number of sequences derived from proteases. We further validated our findings using the web-based tool: Proteasix. It determines confidence thresholds of predicted proteolysis by using MEROPS specificity weight matrices for experimentally confirmed cleavages. MEROPS is a database that provides information about protease substrate sequence specificity in terms of the specificity weight matrix. Based on experimentally confirmed cleavages, the matrix shows the frequency of amino acids at every position of the site. The output data were refined by removing the cleavages that were having specificity below 80%. We determined the common protease activity in all three physiological conditions and found that 54 proteases out of 62 potential proteases (85.7%) were common (Fig. 4A). No protease enzyme could be uniquely associated with pregnancy. However, two unique proteases were reported in the heifer and lactating groups (Fig. 4B). Pearson correlation-based group-wise comparison (heifer, pregnant, and lactation) is 0.99 among each other, describing highly correlated data. The heat map represents the total number of target peptides identified in the data to specific protease (Fig. 4C) and similarity Pearson comparison (Fig. 4D). The occurrence of Matrix Metalloproteases (MMPs) isoforms was wide spread across all the physiological states which were mapped to their target proteins (Fig. 4E). Several isoforms of KLK enzymes were identified with potential cleavage sites on the target proteins leading to the formation of bovine urinary degradome (Fig. 4F). Protease wise comparison and measure of Pearson correlation (Fig. 4D) identified eight different protease enzyme (CASP2, CASP8, FURIN, LGMN, MMP10, MMP17, PCSK1, and PRSS3) that were in disagreement with physiological state and uncorrelated with one another (Fig. 4G). The profile plot and heatmap representing the number of protease targets in all three stages revealed that enzymatic degradation of target proteins during pregnancy is somewhat slow and suppressed (Fig. 4F and A).

### Chromosomal mapping of stage-specific bovine endogenous urinary peptidome

**Figure 5:**
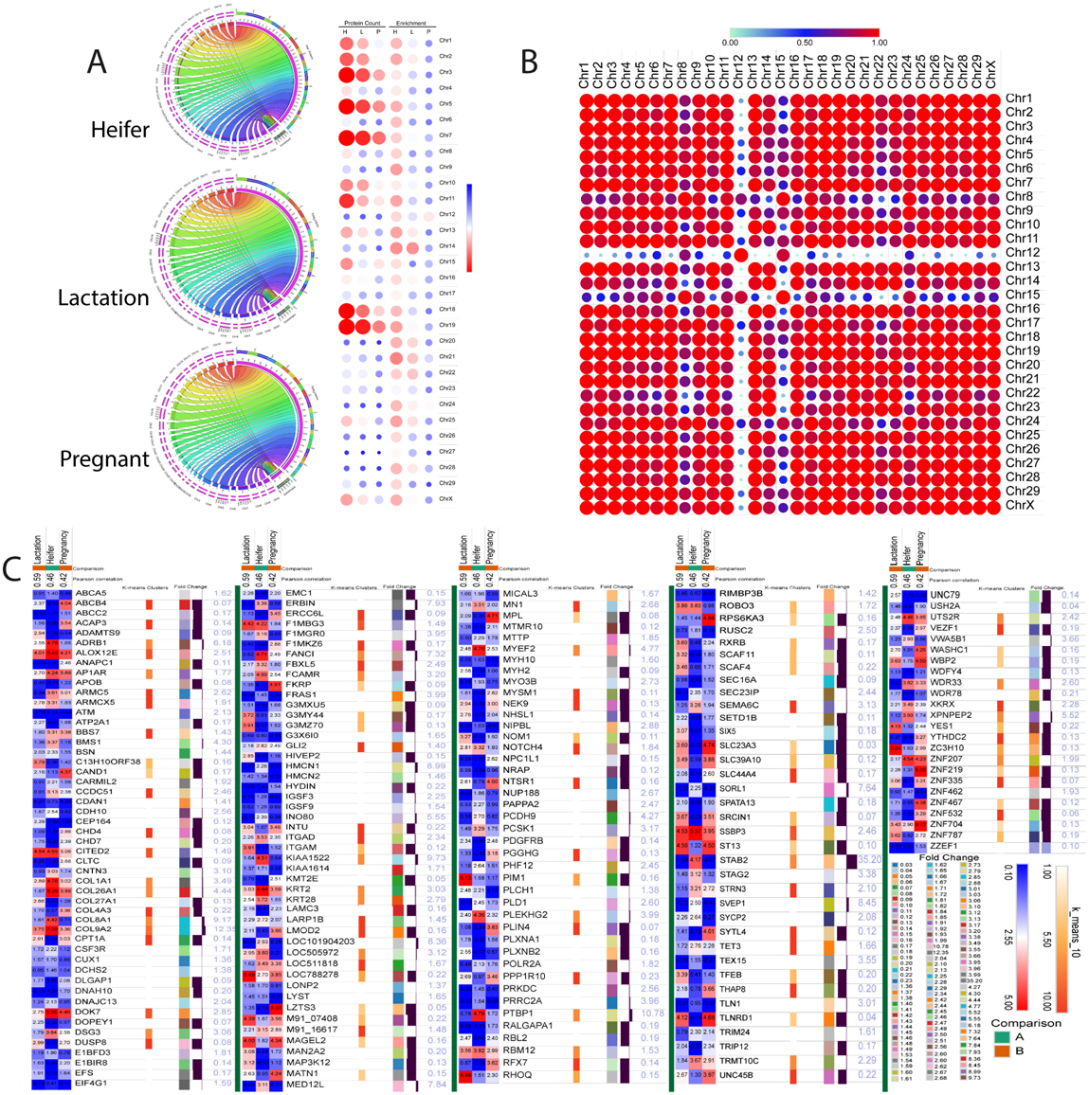
Peptide to protein mapping: (**A**) Circos plots describing the mapping of peptides to specific chromosomes through parent protein. The right-hand side details the information of protein count heat map and enrichment of in three stages. (**B**) Pearson correlation-based similarity matrix plot of all chromosomes and associated target proteins determined with respect to endogenous peptides. (**C**) Heat map presentation of proteins quantitative data for common parental proteins among three stages. Fold change is calculated using the heifer as the control for lactation and pregnancy. Proteins were grouped based on the k-mean clustering and fold change calculate with respective color coding.

Total spectra and protein information identified from the search engine using the UniProt database were represented as the interconnecting bars in the circle (Fig. 5A). Notable results are the identification of the denovo peptides mapped to all the 29 + X chromosome. Interesting facts uncovered was the uneven distribution of the protein expression from the chromosomes in a physiological stage-specific manner. The expression of proteins from the chromosomes was drastically inconsistent at different physiological states (Heifer, Lactation, and Pregnant). Bubble plot-based heat map analysis showed the total protein counts represented from respective individual chromosomes (Fig. 5A, left side) and percentage contribution of the proteins in terms of enrichment from the total number of proteins to the coded in the bovine genome (Fig. 5A, Right side).

Chromosomes 3, 5, 7, 11, 18, and 19 were responsible for hosting the maximum number of endogenous peptides on the genome, whereas chromosomes 12, 20, 24, 27, and 28 gave rise to a minimum number of peptides that are identifiable in the urine. The unbiased analysis of enrichment percentage for all the chromosomes mapped sequences showed the high enrichment of respective peptides from chromosome 14, 20, 21, 22, 25, and 28 in the stages of heifer and lactation. Interestingly, chromosome 12 was rich in the release of peptides in pregnant animal urine samples, whereas all other chromosomes contributed equally. The same results were obtained from the distance-based correlation analysis among chromosomes. We found the distinct expression values of chromosome 8, 12, and 15 in comparison to others (Fig. 5B). Our finding raises a question: what are the reasons responsible for the high expression from certain chromosomes in heifer and lactation stages while the low contribution of coded peptides and proteins from the same chromosomes in the pregnant state? Hitherto, factors that decide the differential expression of chromosomes in different physiological states is not known. We suggest that physiology specific expression of proteins is probably the reason which dictates the profile of peptides in urine. Nonetheless, further studies on genome-wide proteome analysis are required to answer these questions. The heatmap analysis for 483 common parental proteins mapped in all three stages with significant St Peter’s values showed the clustering pattern among the proteins as defined by the Kmean clusters (Fig. 5C). Keeping the heifer stage as the control, we determined the fold change difference among the stages. The results showed the identification of five proteins which are more than 9 fold up-regulated in pregnant and lactation stages, the proteins are STAB2 (FC: 35.20), KIAA1522 (FC: 9.73), COL8A1 (FC: 9.17), COL9A2 (FC: 12.35), and PTBP1 (FC: 10.78). It shows the importance of these five proteins in pregnancy and lactation.

### Tracing back Peptidome to physiology specific functional annotation

**Figure 6:**
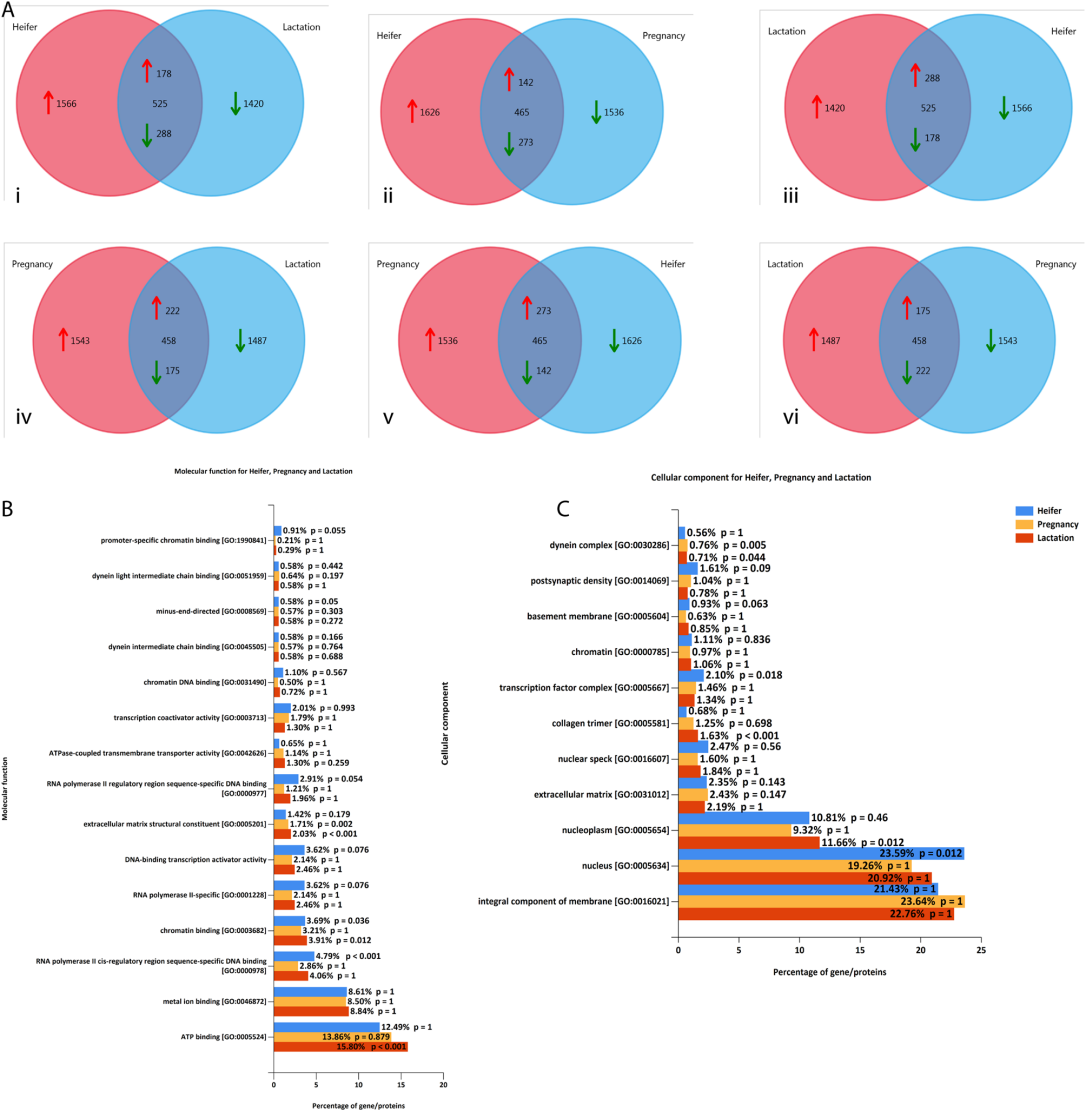
Parental proteome comparison: (**A**) Venn diagram comparison. i) Heifer versus lactation, ii) Heifer versus pregnancy, iii) Lactation versus heifer, iv) Pregnancy versus lactation, v) pregnancy versus heifer, vi) Lactation versus pregnancy. (**B**) Combined molecular function analysis of three conditions. (**C**) Cellular component analysis.

We compared all of the protein concentrations identified through StPeter among the physiological states (Fig. 6A). The analysis identified the 525, 465, 458 common proteins between heifer versus lactation, heifer versus pregnant, and pregnant versus lactation, respectively. The total numbers of average 1500 proteins were identified uniquely in all three conditions. The molecular function analysis showed the identification of parental proteins contributing to the multiple cell replication process (Fig. 6B) with respective p values reported (Supplementary Table 4). The processes are RNA polymerase DNA binding, ATP binding, promoter-specific chromatin binding, minus-end-directed, transcription co-activators. Even though the highest number of proteins corresponds to the heifer, but we found the lactation state showed the high enrichment of the ATP binding process (GO: 0005524) with p-values < 0.001 and 15.80% of involved proteins. However, RNA polymerase activities (GO: 0000978; GO: 0001228; GO: 0000977) in heifer was significant with p-values < 0.001 and proteins involvement of 4.79%. All the pregnancy-specific molecular function ontologies have non-significant p-values except the extracellular matrix structural constituent (GO: 0005201). It is also significant in lactation with p-values<0.001. The cellular component analysis showed the identification of the different class of organelles and cellular parts in the ontological information with respective p-values (Fig. 6C). Again, we found a significant p-value <0.001 in the lactation stage for collagen trimers (GO: 0005581). Altogether these results support our finding for the crucial importance of collagen proteins specific endogenous peptides secretion during pregnancy and lactation in which extensive remodeling of tissue and organs takes place.

**Figure 7:**
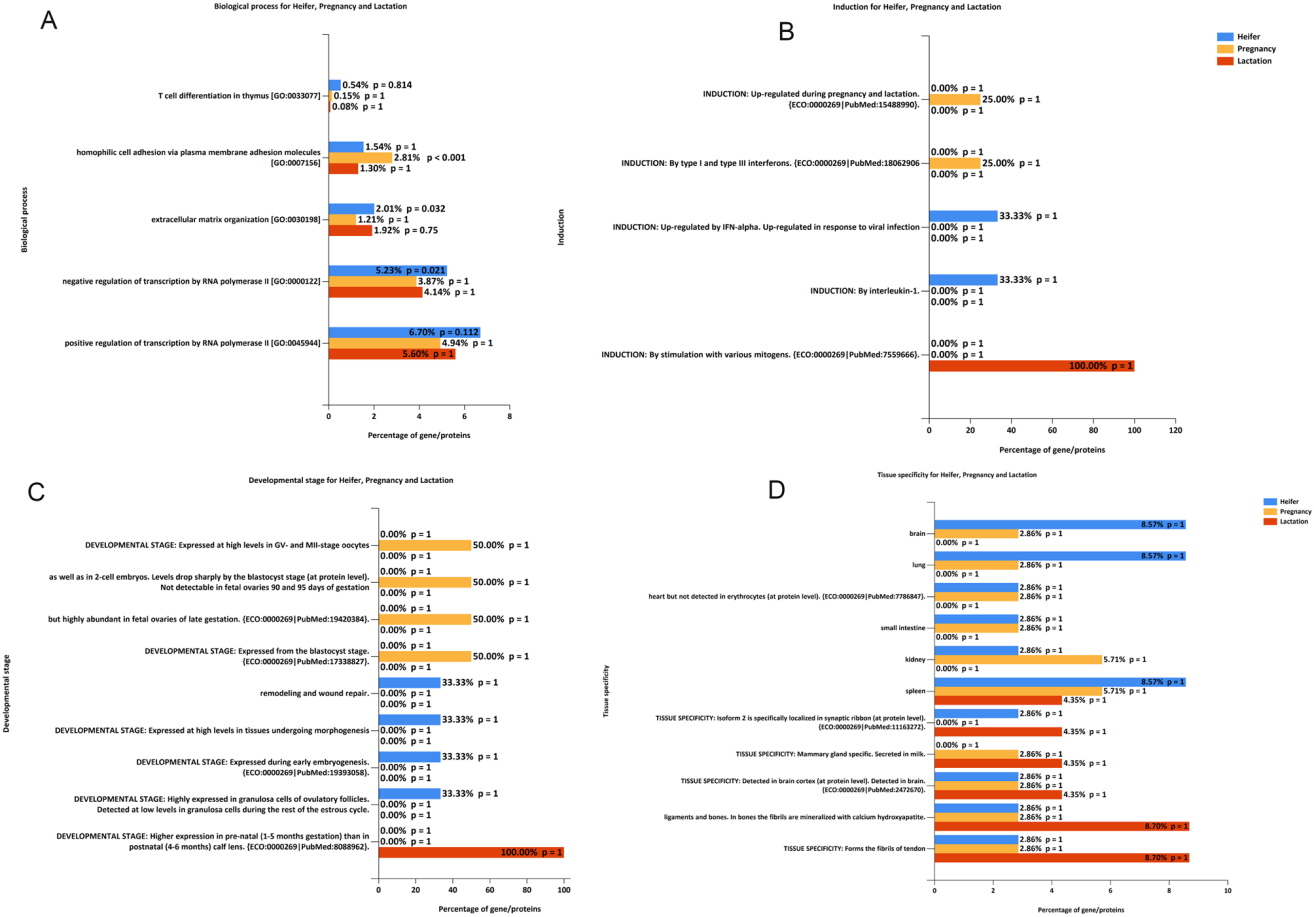
(**A**) Biological process analyses (**B**) Induction of proteins respective to specific conditions. (**C**) Developmental proteins analysis in all three conditions. (**D**) Tissue-specific proteins analysis in three physiological conditions.

Furthermore, the comparison of all three stages for the biological process identified the five most enriched ontologies (Fig. 7A). In correspondence to CC and MF process, we found the identification of extracellular matrix organization (GO: 0030198) and regulation of RNA transcription process (GO: 0000122; GO: 0045944). However, homophilic cell adhesion (GO: 0007156) and T cell differentiation (GO: 0033077) are the unique ontologies determined. Only pregnant specific ontology in homophilic cell adhesion was determined to be significant (p<0.001). Next, we searched our list of proteins from different stages with the induction process (Fig. 7B).

We next sought to retrieve information from the urine proteome by comparing various database based on developmental stage and tissue specificity (Fig. 7C and D). We found the identification of the relevant proteins in the urine specific to the stages previously reported in the literature. The results strongly suggest that the diversity of endogenous peptides is indicative of the physiological state.

## Discussion

The composition of urine is an imperative indicator of the physiology of the organism and hence, can be used as an excellent non-invasively collected biofluid for diagnostic purposes. Endogenous urinary peptides have an obvious edge over full-length proteins as the formers get easy access to urine and at the same time can insinuate toward a particular protein that is disturbed within the body. The study reports a comprehensive profile of endogenous peptides in bovine urine across various physiological states and discusses their biological activities, chromosomal mapping, and molecular features. To date, this is the largest comprehensive bovine urinary peptidome dataset signifying its importance in physiology.

The urinary bladder serves as a temporary reservoir of urine. The protease activity is an essential requirement for continuous turnover of proteins in organs like urinary bladder, uterus or mammary gland wherein muscular elasticity is needed for continuous expansion and contraction. These proteases function in an interactive manner where multiple target proteins interact with multiple proteases of various families and classes creating a protease web^19^. Many of these proteases can be traced back to the urinary bladder which is highly stretchable. However, it is not certain whether these peptides get access to urine through cellular exudates or systemic circulation. A large number of peptides are breakdown products of large proteins involved in urine storage and voiding viz. collagen.

The molecular size distribution of the urinary peptides follows a bell-shaped curve which indicates that the majority of the peptides present in urine are between 700 to 4799 Da. Our study focuses on identification of small sized endogenous peptides (<10 kDa), by scanning MS spectra ranging from 200 to 2200 m/z, so, a fraction of very large and very small peptides might not have been covered in the study.

For peptides to get access to urine through glomerular filtrate, the permissible size cut-off is a critical determinant on the length of urinary peptides found in the urine. Of course, reabsorption in the urinary tract may affect the size distribution^20^. In this case, it seems that most of the peptides are released into the urine because of its excess in the circulation over and above the tubular saturation limit. The observation of several peptides in urine which cannot be attributed to local organs like UB, ureter, urethra, local glands, etc is suggestive of its origin being glomerular filtrate from the blood. For example beta casein isoform and Complement C4-like isoform X1, proteins are mostly found in mammary gland and blood cells respectively^21^. The prevalence of specific peptide sequences over others suggests the crucial role executed by their precursor proteins in a specific physiological context.

To translate the results, we characterized protease substrate cleavages as net outcomes of complex proteolytic activities in normal vis-à-vis diseased physiology to foster the characterization of numerous peptide-binding receptors (PBRs) as potential drug targets. The result suggested that endogenous peptides possess a variety of bioactive functions. Prediction platforms were used to gain a comprehensive insight into the peptide sequence characteristics. We found a pattern of amino acid residues in the sequences that might be contributing to the different types of activities associated with the peptides. Many web-based prediction platforms use the Amino Acid Composition (AAC) based algorithm and literature supported the bioactivity of peptides. Searching for common residues and motifs on peptide may help us to explain the compositional biases of peptides for certain amino acid residues and various beneficial effects attributed to the associated bioactivity. Additionally, functional assays must be performed to support the in-silico results as these algorithms might not be fully accurate.

The cow urine has been granted US Patents for its medicinal properties, particularly as a bio enhancer along with antibiotics, antifungal and anticancer drugs (6896907, 6410059). Jain et al. studied the effect of cow urine therapy on various types of cancers in the Mandsaur area In India wherein they reported that the severity of symptoms (pain, inflammation, burning sensation, difficulty in swallowing, and irritation) subsided significantly^22^. The study identifies a large number of peptides having sequence motifs unique to anticancerous properties. The Pro residue can be considered a common residue on the peptides from two categories namely anticancer and antihypertensive peptides. Scanty information is available on anticancer and antihypertensive peptides. A report determined the possible role of Ang II in tumor progression, as antihypertensive peptides target angiotensin-converting enzyme, they might be a potent anticancer agent^23, 24^.

Many antimicrobial peptides exhibit anti-inflammatory features, the reason why we get a relatively large number of shared sequences between these two sets^25, 26^. Wei *et al.* pointed out that many AMPs are capable of binding to LPS and this might be a possible reason for how AMPs exhibit anti-inflammatory activity by inhibiting LPS induced NO release, a pro-inflammatory mediator^27^.

Sequence analysis showed that Gly was a common and most abundant residue in all classified peptides. Five amino acids namely Gly, Ser, Ala, Leu, and Pro in decreasing order of abundance constitute the major percentage (roughly about 50%) of all residues present in urinary peptides while in an estimate drawn from yeast proteome (6225 known and predicted proteins) it has been reported that four amino acids leucine, serine, lysine, and glutamic acid are the most abundant amino acids, totaling 32 percent of all the amino acid residues in a typical protein^28^. Thus, it can be understood that the urinary peptides are more selective than being random. Shoombuatong *et al.* reported that Gly is the frequently occurring residue in antibiofilm peptides (ABPs), anticancer peptides (ACPs), antifungal peptides (AFPs), antiparasitic peptides (APPs), and antiviral peptides (AVPs) at 10.98%, 10.88%, 10.79%, 10.77%, and 11.82% respectively^29^.

ACE inhibitor peptides are reported to possess aromatic amino acids such as tryptophan, tyrosine, or proline at C-terminal^30, 31^. Consistent with the predicted list of peptides, an adjacent proline residue to C-terminal proline residue also increases the ACE inhibitory activity of peptides^32^.

Anti-inflammatory peptides (AIP) exert their effects by a variety of mechanisms *viz.* inhibition of JAK-STAT and NF-kB pathways, inducing the secretion of IL-4, inhibition of LPS induced cytokines^33–36^. Studies have reported that hydrophobic residues like phenylalanine and leucine have a major influence on the anti-inflammatory effect of peptide^37, 38^. Manavalan *et al.* reported that average composition Arg, Leu, and Lys, were dominant in AIP^39^. Also, Leu alone as a residue has been shown to exert anti-inflammatory activity by reducing the LPS induced NO, a pro-inflammatory mediator in RAW 264.7 macrophage. In our study, we did found Leu to be a relatively abundant amino acid followed by Gly and Ser residues.

Studies have shown that ACPs are mostly dominated by Gly, Lys, Cys, Phe, Ile, and Trp as compared to non-ACPs^40, 41^. On contrary, in our case, sequence logo creation showed that anticancer peptide sequences are dominated by Pro, Gly, Ser, Leu, Arg residues with Pro dominating at almost every position of the sequence. A study reported that the presence of Pro residue enhances the toxicity of peptides against nucleated cancer cells while simultaneously protecting RBCs from lysis^42^. Just like AMP, Anti Cancer Peptide (ACP) also contains cationic residues.The selective killing is mediated through the electrostatic interaction between cationic residues and the anionic membrane of cancer cells^43^. In our finding, Arg residue was the only abundant cationic residue followed by histidine. As already mentioned, collagen-derived peptides constitute a major portion of cow urinary peptides, therefore, finding Pro residue in significant abundance in the case of AHTPs and ACPs is not surprising. The wide spectrum of bioactive properties of cow urine may be associated with the small size of endogenous peptide.

Anti-Microbial Peptides are usually short (10 to 50 amino acids) with an overall positive charge (predominantly +2 to +9) and are present in every form of life^44^. AMPs/ ABPSs exploit the difference between the composition of the prokaryotic and eukaryotic membranes. Lipids in the outer leaflet of the animal cell contain no net charge, while prokaryotic membranes are rich in anionic phospholipids, hence, it is selectively targeted by AMPs^45^.

Peptide sequences were also evaluated based on their physicochemical properties as a fine balance between charge and hydrophobicity drives the activity of the peptide. In agreement with our findings, one study finding showed that most of the position inAMPs was dominated by Arg, Lys, Leu, and Gly^46^. The balance between a positive charge and hydrophobic residue drives the activity of AMP. Positively charged residues in AMP undergo electrostatic interaction with the negatively charged prokaryotic membrane, while hydrophobic residues help the AMP penetration and disruption of the bacterial membrane^47, 48^. Chang *et al* reported that a higher percentage of glycine and lysine was present in the core of AMP and the critical region of AMPs contained glycine as the most abundant residue^49^.

The presence of collagen peptides in the urine is probably due to excessive bladder activity. Several studies reported its role in urinary bladder compliance, proliferation, and bladder filling^50, 51^. A study shows that the ratio of type III to type I collagen determines changes in compliance in both developing fetus and mature bovine UB. Significant reduction in volumetric densities of type I and III collagen was observed with the aging of the urinary bladder in Wistar rats. The urinary bladder and other parts of the urinary system always remain in dynamic states of distension and relaxation to process and accommodate the varying volumes of urine. To ensure this, urinary epithelium and stromal cells undergo extensive extracellular matrix (ECM) degradation and remodeling. ECM epitopes, a direct resultant of MMP mediated proteolysis, are involved in the stretch-induced proliferation of bladder smooth muscle cells through ERK1/2 signaling activation^52^. Collagen being a connective tissue is the major target in the ECM degradation and remodeling process and hence shows its signature in urine in the form of various small-sized EPs. Reinforced by other reports, our finding shows that collagen-derived peptides are abundant in urine^10, 53, 54^ but the individual sequences of the peptides were different in different physiological states. One study found a significant correlation of the relative abundance between urine and plasma samples and hypothesized that collagen-derived peptides, unlike any other peptide in plasma, by an unknown mechanism, escapes tubular reabsorption^54^. In urine, the peptide degradation is least in comparison to other biological fluid like blood serum or plasma^55^. Thus, making it a suitable biological fluid for proteome-based study.

The role of proteases is important for the complete turnover of proteins in various physiological and diseases condition. Approximately, 5–10% of drugs target these proteases^56^. Most of the peptides in the biological fluid are a consequence of proteolytic cleavage of proteins by diverse types of proteases. The disturbance in the protease network leads to various diseases including cancer, cardiovascular, inflammatory, and gastrointestinal disorders^57–59^. However, so far technical limitations have prohibited a global understanding of interconnected protease activity in complex pathophysiological situations. Since pre-pregnancy (heifer), pregnancy and lactation are distinct physiological states; we traced the urinary peptide profile back to the systemic changes in the protease network with a view to identifying any significant correlation between specific protease and physiology. A significant finding was the kallikrein and matrix metalloproteinases class of proteases were actively involved in proteolytic events. MMP class of proteases serves diverse roles; most of them are associated with collagen degradation, cell migration, cancer progression, collagen affinity enhancing, pro and anti-inflammatory activities, etc. Proteases like plasmin, trypsin, chymase, and certain other metalloproteinases viz. MMP-1, MMP-2, MMP-8, MMP-9 are involved in the activation of MMPs^60^.

We identified peptides of Matrix Metallo Proteases (MMP proteases) in urinary peptides (Heifer: MMP-2, 9, 11, 13, 16, 0, 23, 25, 27, 28; Lactation: MMP-9, 16, 17, 23, 24, 25; Pregnant: MMP-8 & 20), which supports the proposed role of MMPs in ECM remodeling of genito-urinary tract and explains the origin of collagen-derived peptides in urine.

Besides MMPs, cathepsin proteases were also predicted with quite a significant number of cleavages and we also found their signatures in MS/MS data. Cathepsins are lysosomal enzymes which include serine, cysteine, and aspartic proteases, just like MMPs, these are also involved in the remodeling of ECM^61, 62^.

Interestingly, during pregnancy, the resultant peptides from protease cleavage were significantly less as compared to the other physiological states. Similar observation was made in the study in mice where the activity of certain proteases such as MMP2 and MMP9 is suppressed during pregnancy, which increases after parturition and reverts to normal^63^.

Molecular Function ontology: extracellular matrix structural constituent (GO:0005201) was found significant in the lactation and pregnancy indicating tissue remodeling during these stages. COL8A1 gene was found upregulated during tissue remodeling in pregnant myometrium (human)^64^. During mammary cell growth in bovine, COL8A1 is involved in epithelial cell proliferation which was found to be upregulated during lactation^65^. Homophilic cell adhesion (GO: 0007156) was found to be significant in pregnancy stage which plays significant role during embryo implantation^66, 67^.

Searching list of proteins from different stages with induction process showed that none of the processes was found to be shared in all three stages; however, proteins associated with specific processes were explicitly relevant to the physiological condition. We identified the GLUT8 peptides in pregnant animal urine, which was upregulated during the pregnancy induction process. GLUT8 mRNA expression in the bovine mammary gland was increased 10-fold (P<0.001) during late pregnancy and early lactation^68^.

## Conclusions

We presented a simple method for the discovery of thousands of endogenous peptides in cow urine that contribute to various bioactivities associated with urine. The data presented here represents the identification of  5000 natural peptides in all three physiological conditions laying the foundations for detailed studies. The molecular weight, peptide length, and amino acid distribution of endogenous peptides follow a similar pattern in all three stages. The study emphasized on the identification and characterization of endogenous peptides of <10 kDa molecular weight by scanning MS spectra ranging from 200 to 2200 m/z. which represented peptides of molecular weight ranging from 700 to 4799 Da. As our study used C18 silica gel for the peptide extraction, many of the highly cationic and hydrophilic peptides might not have been covered. A combination of chromatographic methods can still improve the identification coverage of urinary peptides. Several of the bioactive properties associated with the peptides were predicted using in-silico platforms. We provided evidence for the peptide-mediated antimicrobial activity against *E. coli* and *S. aureus* but more experiments are needed to validate other predicted bioactivities. This study also analyzed the complex network of proteases active during specific physiological states and the target proteins as precursors of the urinary peptides. The knowledge about physiology-specific proteins and proteases may generate further interest in the field of biomarker discovery for pregnancy diagnosis or in understanding the pathophysiology during development and lactation. The urinary peptides also represent degradome and explain the formation and release of peptides from precursor proteins by specific proteases. Lastly, the tissue-specific developmental gene ontological classification derived from the protease-targeted proteome highlighted the stages at which specific set of proteins are active in specific physiology. The data opens future avenues to set a benchmark for urine-based biomarkers unaffected by physiology and also helps to understand various functional activities associated with cow urine. The present study has searched for the peptides based on primary sequence molecular weight only. However, it will be interesting to observe and search for post-translationally modified peptides including glycosylated, phosphorylated, and others.

## Methods

### Sample collection

TThe urine samples were collected from 30 healthy female Sahiwal cattle [*Bos indicus*, belonging to three different physiological states namely, heifer (n = 10), pregnancy (n = 10), and lactation (n = 10) in the presence of a veterinary doctor from Livestock Research Centre located at National Dairy Research Institute (NDRI) Karnal. All the animals included in the study were clinically healthy and divided into three groups (Heifer (age between 17th–18th months), pregnant (40th–60th days of pregnancy), and lactating (80th–100th days of lactation). All procedures were approved by the Institutional Animal Ethics Committee (IAEC) ICAR-NDRI, Karnal, India.

Within each group, a sample was created by pooling samples of ten animals (biological replicates n = 10) and processed separately with six technical replicates. However, to understand the animal to animal biological variations, we performed a separate study on seven individual animals from each category. The procedure for the samples collection and processing is the same for all the groups as described here briefly. Approx. 500 mL of fixed time morning voids urine samples were collected by massaging the perineum of the animal manually. The collected samples were immediately transferred to the lab and analyzed for any debris, dung, dust, or hair to rule out any contaminants in the samples. Initially, the samples were filtered by muslin cloth followed by centrifuging at 7000 rpm for 20 min to allow settlement of any cell debris and particulate matter. The microscopic examination was performed for individual samples, before and after the centrifugation, to observe the presence of RBCs, WBCs, other cells, and debris. The purified clean urine was further used for peptide extraction and purification (Fig. 1A).

### Peptide extraction

The clean urine samples were passed through ultra-filtration assembly (Thermo easyload Masterflex, model 7518-00, USA) with a 10 kDa molecular weight cut-off filter (Pall MinimateTM TFF Capsule), to separate the endogenous peptides mixture. The pH of the obtained filtrate was adjusted to ≤3 using Trifluoroacetic acid (TFA) for further treatment. The peptides mixture (PM) was subjected to manually prepared C-18 beads based Solid Phase Extraction columns (SPE) (Fig. 1B). Briefly, the column was prepared using the C-18 reversed-phase silica gel (Sigma, Switzerland Cat. No.60757-50G). A slurry of 20 grams of silica gel was prepared in methanol. The packing of the column was done by continuously stirring the slurry and then slowly draining it into the column followed by several washes with methanol.

The packed column was conditioned using 90% methanol followed by equilibration with 10 column volumes of 0.1% TFA in water. After equilibration, PM was loaded with a flow rate of 0.5 ml/min followed by desalting using 5% methanol with 0.1% TFA. The desalted peptides were eluted in 60% acetonitrile (ACN) with 0.1% TFA. Processing of around 500 mL of urine yielded approx. 30–50 mL of eluate with dark brown appearance. For the removal of the dark brown substances (possible contaminating metabolites in urine) the ethyl acetate-based extraction was performed. The eluates were subjected to the double volume of ethyl acetate followed with end to end rotation for 5 min and allowed to settle for 10 min to differentiate into two layers. The upper organic layer was stored appropriately for metabolome profiling and the lower aqueous phase was aliquoted in 2 mL microcentrifuge tubes and dried by speed vac (Thermo savant ISS110 SpeedVac concentrator, ISS110-230, USA).

The dried samples were stored at $-80\,^{\circ }$C. Prior to mass spectrometer run the routine sample cleanup was performed by Pierce C-18 Spin Columns as per the manufacturer’s protocol. Briefly, the quantified samples were reconstituted in 20% ACN with 2% TFA (sample buffer, 3 µL for every 1 µL of the sample). After loading and washing of the sample, peptides were eluted in 70% ACN with 0.1% TFA. The eluted samples were dried and reconstituted in 0.1% TFA before MS run.

### Electrospray ionization tandem mass spectrometry LC-MS/MS analysis

The reconstituted peptides were used for shotgun proteomics experiments for the identification of the endogenous peptides. The peptides were separated using micro-LC (Dionex, Thermo UltiMate 3000 HPLC System, USA) through analytical column (Supelco, Ascentis Express C18, 25 cm $\times$ 4.6 mm, 2.7 µm) coupled with ESI source (BrukerDaltonics, Germany) spray in Maxis-HD qTOF (Bruker, Germany) mass spectrometer. The acquisition parameters were adapted from our previous reports with slight modifications^69, 70^. The elution was performed with a flow rate of 150 µL/min a continuous gradient of 5–75% acetonitrile over 135 min. In the solvent system; Solvent A was 100% water with 0.1% formic acid, and solvent B was 100% acetonitrile with 0.1% formic acid. Data were acquired in the data-dependent mode in mass spectrometer operated in automatically switching between MS and MS/MS acquisition. The precursor ion MS spectra scan range of 200–2200 (m/z) was used in the Q-TOF with resolution R = 75,000. The six most abundant precursor ions were searched for detection of different masses during acquisition and selected for fragmentation using collision-induced dissociation (CID) with a fixed cycle time of 3 s along with 2 min of release for exclusion filter (otof processing software, BrukerDaltonics).

### Data processing

The .d raw data files were subjected to TPP pipeline for the identification of endogenous peptides. For analysis, the otof generated raw (.d) files were converted to mzML format using MSconvert GUI using the default parameters. The converted mzML files were searched for MS/MS spectra using the Trans-Proteomic Pipeline version 5.1.0 released on 2017-11-03 on in-house combined UniProt *Bos taurus* (Cow), *Bubalus bubalis* (Buffalo), common contaminant sequences, and an equal number of decoy sequences database. The detailed protocol was reported in Suhail et al., 2019^71^. Briefly, for the analysis, the peptide assignments were performed using multiple search engines using X! Tandem (with the k-score plug-in), and Comet. For both the search engine the search parameters included un-digested peptides and the remaining parameters were kept as default. The minimum peptide length parameter was set to 6 amino acid residues. Further Peptide Prophet and Protein Prophet algorithms were used in the pipeline to compute the probabilities score for both individually searched peptides and the respective proteins. The accurate mass model in Peptide Prophet was used for high confidence peptide identifications to boost the probability of peptide identification. Another protein validation step was executed using both Peptide Prophet and Protein Prophet Scores, where the protein was authenticated if it contained a minimum of two top-ranked peptides with each peptide probability score above 95%. All the search engine results were merged and validated using iProphet. This method takes the input of Peptide Prophet spectrum-level results from multiple LC-MS/MS runs and then computes a new probability at the level of a unique peptide sequence (or protein sequence). This framework allows for the combination of results from multiple search tools and takes into account other supporting factors, including the number of sibling experiments identifying the same peptide ions, the number of replicate ion identifications, sibling ions, and sibling modification states. A model of iProphet performance concerning the number of correct identifications versus error. An iProphet probability of more than 0.95 was used as the cutoff for the final identification of the protein. For protein quantitation, the MS2-based Normalized Spectral Index (SIN) was used in the StPeter algorithm implemented in TPP for all proteins identified with $\ge$2 unique peptides per protein.

### Antimicrobial assay

The total urinary peptide from all three groups extracted by SPE was then assessed for its a AntiMicrobial Activity (AMA). To determine its AMA the peptide was reconstituted in milliQ water and coated on 6 mm sterile discs (HIMEDIA, SD067-1VL). The discs were allowed to dry under a laminar hood. A 0.5 McFarland equivalent of test cultures (*Staphylococcus aureus* ATCC 29213, *Escherichia coli* ATCC 25922) were swabbed on the surface of Mueller Hinton Agar (HIMEDIA, GM173-500G) and allowed to dry. With the help of sterile forceps, the coated discs were placed on the lawn of the test culture and incubated overnight. BSA digest was used as the negative control in the experiment. The appearance of the inhibition zone confirms the antimicrobial activity of the cow urinary peptides derived from all physiological states.

### Bioinformatics analysis

All the graphical analyses were performed in the R environment using the ggPlot2 package^72^. The Gene Ontology (GO) categories were analyzed using the DAVID bioinformatics resources and only the genes with an adjusted P-value (false-discovery rate) of less than 0.05 were included for subsequent GO term plotting.

### Strategy for manual and MERPOS based protease prediction

Peptide list belonging to different physiological states of cows was sorted based on possible protease cleavage site located on four residues either from N or C terminal. The hypothesis was to identify the protease responsible for its release from the precursor sequence. The peptide sequences were traced back to 21 possible proteases, which provided hints to explain the physiology driven variation in the number and type of sequences derived from each physiological state.

Proteasix tool was used for the prediction of cleavage sites which uses the MEROPS peptidase database as a reference. It provides specificity weight matrices for experimentally validated cleavages for proteases. The batch peptide match tool from Protein Information Resource (PIR) was used to retrieve the start and end amino acid position from the peptide sequence. The retrieved information from PIR was used as an input for Proteasix. Cleavages predicted with more than 80% specificity were selected for the heat map analysis. The aforementioned method was used to predict the cleavages and the associated proteases for heifer, lactation, and pregnant groups.

### Characterization and classification of bioactive peptides based on machine learning SVM model

In-silico analysis was done using web-based platforms to identify bioactive peptides present in cow urine. For validation purposes, the online activities predicting servers using Support Vector Machine (SVM) based algorithm were used. SVM, an established machine learning tool has been extensively used to design prediction platforms for the classification of various kinds of bioactivities associated with peptides^73–76^. Peptides sequences associated with anticancer, anti-inflammatory (AIP), antihypertensive, antimicrobial, and antibiofilm activities were predicted with a high confidence score at or above a higher threshold value of 0.9. Peptides with ACE-inhibitory effect of antihypertensive activity were obtained from antihypertensive peptides inhibitors (AHTpin) (http://crdd.osdd.net/raghava/ahtpin/). The database consists of 1745 entries as a positive dataset from various sources *viz.* AHTPDB, BIOPEP, ACEpepDB, and literature whereas negative datasets consist of a random fragment of proteins of the same length from Swiss-Prot^75^. Sequence logo was designed for the predicted sequence retrieved from AHTpin to determine the abundance of amino acid residues at the C-terminus of the peptides as the presence of certain amino acid residues has a significant impact on antihypertensive activity. For the prediction of AIPs, a web server anti-inflam (http://metagenomics.iiserb.ac.in/antiinflam) was accessed.

Anticancer activity was predicted using the tumorHPD server (http://crdd.osdd.net/raghava/tumorhpd/). This tool utilizes 651 experimentally validated peptides (peptides binding to tumor) in a positive dataset and 651 non-tumor binding peptides randomly generated from proteins obtained from SwissProt74.

Two servers CAMPR3 (http://www.camp3.bicnirrh.res.in/campHelp.php), a database containing entries of antimicrobial peptides from various sources and the other dPABBs which uses SVM based algorithm for the prediction of Anti Biofilm Peptides (ABPs) (http://ab-openlab.csir.res.in/abp/antibiofilm/) were used to predict antimicrobial activity associated with the urinary peptides. Peptides exhibiting antibiofilm activity on at least 4 prediction models were selected. The tool uses a positive dataset consisting of 90 AMPs that are proven through in vitro and in vivo reports to be active against biofilms and 88 Quorum sensing peptides constitute negative dataset. The prediction is based upon whole amino acid composition, features of the residues, and their positional preferences^76^.

### Chromosomal mapping of urinary peptides

All the identified proteins were mapped with Uniprot bovine chromosomal proteome information database (*Bos taurus* taxid 9931) and parsed to create files appropriately formatted for input to Circos for circular visualization and annotation^77^.

### Ethical approval and consent to participate

The approval for the experiment was obtained from Institutional Animal Ethics Committee (IAEC) of National Dairy Research Institute (IAEC approval no. 41-IAEC-18-50). Methods were carried out in accordance to IAEC and ARRIVE guidelines. The urine samples were collected from the Livestock Research Centre (LRC) of National Dairy Research Institute (NDRI), Karnal which is a public funded research institute under the Indian Council of Agricultural Research, Government of India. The second morning void urine samples were collected from three different physiological states of Sahiwal cow. The animals were maintained under expert veterinary supervision. NDRI has all the necessary permits for the housing and care of animals for scientific purposes vide registration no. 1705/GO/ac/13/CPCSEA 3rd July, 2013 duly approved by Ministry of Environment and Forest, Govt. of India (Web site: http://envfor.nic.in).

## Supplementary Information

Supplementary Information.

Supplementary Information.

Supplementary Information.

Supplementary Information.

Supplementary Information.
